# Network characteristics of a referral system for patients with hypertension in Western Kenya: results from the Strengthening Referral Networks for Management of Hypertension Across the Health System (STRENGTHS) study

**DOI:** 10.1186/s12913-022-07699-8

**Published:** 2022-03-07

**Authors:** Aarti Thakkar, Thomas Valente, Josephine Andesia, Benson Njuguna, Juliet Miheso, Tim Mercer, Richard Mugo, Ann Mwangi, Eunice Mwangi, Sonak D. Pastakia, Shravani Pathak, Mc Kinsey M. Pillsbury, Jemima Kamano, Violet Naanyu, Makeda Williams, Rajesh Vedanthan, Constantine Akwanalo, Gerald S. Bloomfield

**Affiliations:** 1 Duke University School of Medicine, 300 West Morgan Street, Durham, NC 27701 USA; 2 University of Southern California, Los Angeles, CA USA; 3 Academic Model Providing Access to Healthcare (AMPATH), Eldoret, Kenya; 4 Moi Teaching and Referral Hospital, Eldoret, Kenya; 5 The University of Texas at Austin Dell Medical School, Austin, TX USA; 6 College of Health Sciences, Moi University, Eldoret, Kenya; 7 College of Pharmacy, Purdue University, West Lafayette, IN USA; 8 Icahn School of Medicine at Mt. Sinai, New York, NY USA; 9 University of California San Francisco School of Medicine, San Francisco, CA USA; 10 National Heart, Lung and Blood Institute, Bethesda, MD USA; 11 New York University, Grossman School of Medicine, New York, NY USA

**Keywords:** Hypertension, Referral patterns, Network analysis

## Abstract

**Background:**

Health system approaches to improve hypertension control require an effective referral network. A national referral strategy exists in Kenya; however, a number of barriers to referral completion persist. This paper is a baseline assessment of a hypertension referral network for a cluster-randomized trial to improve hypertension control and reduce cardiovascular disease risk.

**Methods:**

We used sociometric network analysis to understand the relationships between providers within a network of nine geographic clusters in western Kenya, including primary, secondary, and tertiary care facilities. We conducted a survey which asked providers to nominate individuals and facilities to which they refer patients with controlled and uncontrolled hypertension. Degree centrality measures were used to identify providers in prominent positions, while mixed-effect regression models were used to determine provider characteristics related to the likelihood of receiving referrals. We calculated core-periphery correlation scores (CP) for each cluster (ideal CP score = 1.0).

**Results:**

We surveyed 152 providers (physicians, nurses, medical officers, and clinical officers), range 10–36 per cluster. Median number of hypertensive patients seen per month was 40 (range 1–600). While 97% of providers reported referring patients up to a more specialized health facility, only 55% reported referring down to lower level facilities. Individuals were more likely to receive a referral if they had higher level of training, worked at a higher level facility, were male, or had more job experience. CP scores for provider networks range from 0.335 to 0.693, while the CP scores for the facility networks range from 0.707 to 0.949.

**Conclusions:**

This analysis highlights several points of weakness in this referral network including cluster variability, poor provider linkages, and the lack of down referrals. Facility networks were stronger than provider networks. These shortcomings represent opportunities to focus interventions to improve referral networks for hypertension.

**Trial registration:**

Trial Registered on ClinicalTrials.gov NCT03543787, June 1, 2018.

**Supplementary Information:**

The online version contains supplementary material available at 10.1186/s12913-022-07699-8.

## Background

Hypertension is a leading risk factor for cardiovascular death [1]. Approximately 80% of deaths due to cardiovascular disease (CVD) such as stroke or ischemic heart disease, occur in low-and-middle income countries (LMICs) [2]. This disproportionate burden is in large part due to low overall awareness, treatment, and control of hypertension in these countries, despite the availability of low-cost treatment options [3–6]. A national survey on hypertension in Kenya found the prevalence of hypertension to be approximately 25% (95% confidence interval [CI]; 22.6–26.6%); however, only 15.6% (95% CI; 12.4–18.9%) of these individuals were aware of their diagnoses [7]. Of the individuals aware of their hypertension, 26.9% (95% CI; 17.1–36.4%) were on treatment, with adequate blood pressure control in only 51.7% (95% CI; 33.5–69.9%) [7].

Many health systems and practices in LMICs have been structured and financed to address acute illness; however, an integrated approach to chronic disease care requires attention to the unique resources, coordination, and follow-up required for optimal outcomes [8]. The inability of patients to receive appropriate screening, referral and hypertensive control highlights a health system failure and greater need for health care delivery practices that can appropriately address the complex contributing factors and comorbidities of chronic diseases such as hypertension [9–11].

Effective referral networks have proven to be cost-effective and successful in achieving better health care delivery for chronic diseases such as CVD and human immunodeficiency virus (HIV) in high-income countries as well as LMICs [12–15]. Many LMICs, including Kenya, have referral network recommendations from the central government applicable to community, primary, secondary, and tertiary level facilities, with escalating care options offered at each higher level [16, 17]. However, a number of barriers to successful referral completion continue to persist in LMICs; including cost of medical care, physical transportation and waiting time [18–23]. A successful referral includes the initiation of referral, the movement of the patient to a higher or lower level of care, and evaluation of the patient by the provider at the receiving facility. In Western Kenya, all providers undergo regular training on hypertension referral guidelines, and patients are often given referral cards to assist with transfer of information. However, referral processes and completion rates remain variable across this region.

The Strengthening Referral Networks for Management of Hypertension across the Health System (STRENGTHS) study is a cluster randomized trial aimed at improving hypertension control and reducing CVD risk by strengthening referral networks. The study aims are to (1) conduct a baseline needs assessment to better examine existing referral patterns, gaps, and opportunities for patients with hypertension; (2) use human centered design [24] to plan and launch an intervention to improve referrals; and (3) examine the effectiveness and cost-effectiveness of the intervention with respect to blood pressure control and CVD risk reduction [25].

Network analysis is an ideal methodology to understand existing referral patterns, gaps, and opportunities for patients with hypertension with particular attention to the referral system. Social network analysis is the study of relationships between people and groups and the influences of these connections on behavior [26, 27] and has been applied to health services research to help understand, explain, and change behavioral patterns and disease spread [28, 29]. Social network analysis can be egocentric (focused on individuals and their relationships and behaviors with direct ties) or sociometric (understanding direct and indirect relationships of all individuals in a network) [30]. We chose sociometric analysis as the methodology best suited to characterize referral network patterns between individual providers as well as between facilities across the entire network.

## Methods

### Study setting

The STRENGTHS study includes nine geographic clusters with 54 sites in western Kenya including primary, secondary, and tertiary care level facilities within the Academic Model Providing Access to Healthcare (AMPATH) program. The AMPATH program is an academic global health partnership between Moi Teaching and Referral Hospital (MTRH), Moi University College of Health Sciences, and a consortium of North American universities, pioneered and led by Indiana University [31–33]. AMPATH was conceived as a means to improve population health and initially focused efforts on providing comprehensive HIV care for a catchment area of over 20 million individuals. In recent years, AMPATH has built upon their successes with HIV care to develop hypertension, cardiovascular and other chronic disease management approaches to address the growing burden of non-communicable diseases in Kenya, where less than 20% of those with hypertension are aware of their diagnosis and only 27% are on treatment [34, 35]. This study was conducted in the localities of: Bunyala, Burnt Forest, Busia/Kocholya, Butula, Kitale/Trans Nzoia, Mosoriot/Nandi, Turbo/Uasin Gishu, Webuye/Bungoma, and the clinics associated with Moi Teaching and Referral Hospital in Uasin Gishu which are home to AMPATH chronic disease management (CDM) clinics and the institutions to which they patients receive hypertension care across different health facility levels as appropriate. Referrals to higher level and lower level facilities are referred to as “up-referral” and “down-referral,” respectively throughout this paper.

### Participants

We performed site visits at each CDM clinic to coincide with regularly scheduled clinic days. At each site, only providers who provided care and were involved in decision-making for patients with hypertension were eligible and recruited to be a part of the referral network analysis. These providers included some nurses, clinical officers (similar to advanced practice providers), medical officers, and physicians/consultants. We contacted the head nurse or administrator for each clinic prior to our site visit to obtain a list of eligible providers, and we reviewed the list again on arrival adding any newly identified providers not already included and removing individuals who did not meet eligibility criteria. Some 217 individuals were initially screened to be included in the analysis; however, only 165 met eligibility criteria after a thorough review. The strength of this referral network analysis is contingent upon as much representation as possible. Thus, all individuals who directly participated in the care of patients and referrals were included in our study, unless they were unable to be present for data collection*.* Providers who only gathered vitals for patients with hypertension before clinic visits were not eligible to be a part of the study.

### Study tools

We designed a survey to gather demographic and social network analysis data from each individual. The initial draft of the survey was created by the STRENGTHS team after a thorough literature review of social network analysis techniques with feedback and review by a social network expert. The team on the ground provided additional context to help capture the nuance of the Kenyan healthcare system. We used input from all study personnel and made modifications after conducting mock interviews with providers who were not participating in the study directly. We collected basic demographics, work history, education, and clinical experience information. Years worked at current health facility was categorized as 0–1 year, 2–5 years, 6–10 years, 11–15 years, and 16 or more years. Job titles included: nurse, clinical officer, medical officer, and physician/consultant. We categorized degree of education as the highest academic degree received including: Certificate, Diploma, Bachelors, Masters, and Doctorate or higher. We asked providers to estimate the average number of patients with hypertension they see in 1 month. Providers were asked to nominate up to seven (a) individuals to whom and (b) facilities to which they refer both patients with complicated, uncontrolled hypertension and patients with controlled, uncomplicated hypertension. Providers had the option to state if they did not refer patients at all, “None,” or they referred patients but not to a specific provider, “Unspecified.” See Additional file 1 for our social network analysis questionnaire.

### Procedures

We employed structural network analysis to characterize the referral network by administering a survey, as described in *Study tools*. We obtained signed voluntary informed consent from each research participant. Interviews were conducted in English by research assistants who received individualized training on the survey including role-playing. The survey occurred face-to-face and consisted of two parts: (1) participant demographic data and (2) social network interview. The survey lasted approximately 15–20 min. All participants received compensation for their time. Data were entered and managed using the REDCap platform hosted by AMPATH [36, 37]. The baseline data collection occurred from October 2018 to January 2019 for seven clusters. Two clusters were added to our study in April 2019, and baseline data collection for these additional sites occurred in May 2019. All methods were performed in accordance with the relevant guidelines and regulations.

### Data analysis

To assess basic referral patterns of providers, the number of providers who responded “None” or “Unspecified” was calculated as a proportion for both up- and down-referrals across the primary, secondary, and tertiary levels. Degree centrality measures were used to identify which providers in each cluster made and received the most referrals [38, 39]. In-degree centrality represents the number of links (nominations) each provider receives. For example, if five providers stated they referred patients to Dr. X, then Dr. X would have an in-degree centrality score of five. Individuals with high in-degree scores were noted as “influencers.” Betweenness scores are another centrality measure calculated as the frequency a provider lies on the shortest path connecting other providers [39]. Individuals with high betweenness scores were noted as “bridge-makers” between different parts of a network. Betweenness scores were normalized for cluster size.

We performed a mixed-effects regression model to observe the effect of provider characteristics on the likelihood of receiving referrals. The dependent variable in this model was the number of in-degree nominations providers received, while the independent factors were provider characteristics including provider role, facility level, years worked at facility, sex, age, and average number of hypertensive patients seen in a month. Primary level facilities (health centers and dispensaries) were combined to compare to upper level facilities. We further adjusted for geographic cluster variability. Missing continuous data were imputed with the median. Data for up-referrals were best fit with a mixed model Poisson regression to calculate an incidence rate ratio, and the bootstrap method was employed to account for small data size and over dispersion [40]. Due to the limited number of down-referrals, we were unable to determine the predictors of receiving a down-referral even with the use of the bootstrap method.

We tested a core-periphery model to quantitively assess the strength of our referral networks [41–44]. A perfect core-periphery model has a central core of densely connected nodes and a periphery composed of nodes with loose connections to each other and to the core. A perfect core-periphery structure increases network stability and resiliency in the face of real world financial and resource constraints [43, 45]. To quantitatively access their strength, the referral networks for each cluster in this study were fit to a perfect core-periphery model to calculate a core-periphery correlation score (CP). The better the fit, the higher the CP score, such that a perfect core-periphery network would have a CP of 1.0.

Demographic, descriptive, and regression analyses were conducted using StataSE Version 16 and RStudio Version 1.1.456. Core-periphery Models were run using Borgatti and Everett’s core-periphery algorithm in UCInet [41] and network visualizations were generated in Gephi Version 0.9.2 [46]. The Institutional Research & Ethics Committee at MTRH in Eldoret, Kenya reviewed and approved this study, as well as the Institutional Review Board at Duke Health.

## Results

**Fig. 1:**
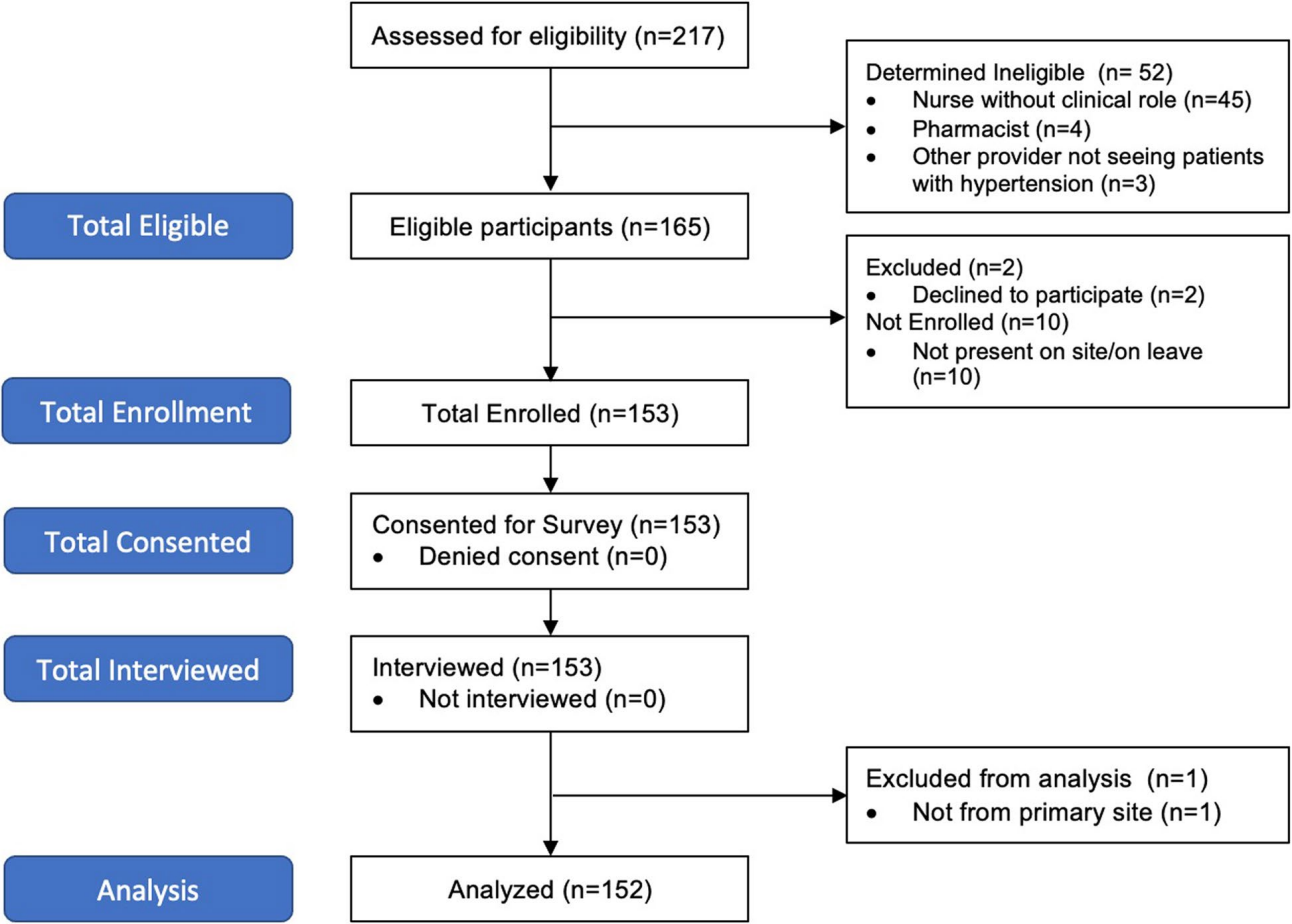
Screening and enrollment diagram

From the nine geographic clusters and 54 facilities, we identified 165 providers eligible for our study. Two providers declined to participate, while the remaining 10 providers were on leave during the site visits and therefore unable to be interviewed. Thus, we enrolled, consented and interviewed 153 providers. Of 153 interviews conducted, one individual self-identified to be from a site not included in our study and was removed from the analysis, leaving 152 total provider surveys for analysis (Fig. 1).

Clusters in Table 1 are listed left to right in order of increasing level of facility (primary to tertiary) and numbers of facilities, from Burnt Forest having only two lower level facilities to MTRH, with three tertiary facilities corresponding to three specialist clinics. Butula, Mosoriot, and Bunyala clusters had approximately equal numbers of clinical officers and nurses, while other clusters were skewed to have a majority of one type of provider. While most providers in each cluster were predominantly male, Kocholya and Turbo were both female predominant and Mosoriot had equal numbers of males and females enrolled. The average number of hypertensive patients seen per month ranged from 42 in Butula to 138 in Burnt Forest; however, given the small numbers of providers and facilities, the variation for all clusters was high (range 1–600, median 40 per month). Beyond facility make up, geographic clusters were heterogeneous in provider characteristics. The majority of participants were clinical officers (*n* = 75), followed by nurses (*n* = 53), medical officers (*n* = 14) and physicians (*n* = 10). Over half of the participants were male (*n* = 84, 55%) and ranged in age from 24 to 61 years with a mean age of 35.7. The clusters were similar with regards to provider work experience and degrees earned. The majority of providers in each cluster had earned diplomas and worked at that facility from 0 to 5 years, with the exception of MTRH, where the majority of providers had earned a Bachelor’s or Master’s degree and been working for greater than 6 years.

**Table 1:** Characteristics of clusters and providers within each cluster

	Burnt Forest	Webuye/Bungoma	Butula	Mosoriot/Nandi	Kocholya/Busia	Bunyala	Turbo	Kitale/Trans Nzoia	MTRH	***P***
**# of Facilities**	**2**	**2**	**5**	**7**	**8**	**9**	**9**	**8**	**3**	
Primary	1	1	4	6	7	8	8	2	0	
Secondary	1	1	1	1	1	1	1	6	0	
Tertiary	0	0	0	0	0	0	0	0	3	
**# of providers (%)**	**10**	**13**	**11**	**10**	**36**	**11**	**24**	**26**	**11**	**< 0.001**
Physician	1 *(10.0%)*	3 *(23.1%)*	0 *(0%)*	0 *(0.0%)*	0 *(0%)*	0 *(0%)*	0 *(0.0%)*	1 *(3.8%)*	4 *(36.4%)*	
MOs	2 *(20.0%)*	1 *(7.7%)*	0 *(0%)*	0 *(0.0%)*	1 *(2.8%)*	0 *(0%)*	1 *(4.2%)*	5 *(19.2%)*	3 *(27.3%)*	
COs	6 *(60.0%)*	9 *(69.2%)*	6 *(54.5%)*	5 *(50.0%)*	11 *(30.6%)*	5 *(45.5%)*	13 *(54.2%)*	18 *(69.2%)*	2 *(18.2%)*	
Nurse	1 *(10.0%)*	0 *(0%)*	5 *(45.5%)*	5 *(50.0%)*	24 *(66.7%)*	5 *(45.5%)*	10 *(41.7%)*	2 *(7.7%)*	2 *(18.2%)*	
Other	0 *(0%)*	0 *(0%)*	0 *(0%)*	0 *(0%)*	0 *(0%)*	1 *(9.1%)*	0 *(0%)*	0 *(0%)*	0 *(0%)*	
**Sex (%)**										**0.03**
Male	8 *(80.0%)*	9 *(84.6%)*	6 *(54.5%)*	5 *(50.0%)*	14 (*39.9%)*	6 *(54.5%)*	11 (*45.8%)*	16 *(61.5%)*	9 *(77.8%)*	
Female	2 *(20.0%)*	2 *(15.4%)*	5 *(45.5%)*	5 *(50.0%)*	22 *(61.1%)*	5 *(45.5%)*	13 *(54.2%)*	10 *(38.5%)*	2 *(18.2%)*	
**Age (SD)**	32.6 (±6.1)	34.3 (±7.8)	38.1 (±9.7)	37.2 (±6.8)	37.1 (±8.67)	35.6 (±9.3)	35.0 (±6.2)	32.6 (±6.7)	41.6(±8.2)	**0.03**
**Avg # HTN pts in 1mo (SD)**	137.5 (±125.0)	70.4 (±60.9)	42.2 (±60.4)	53.5 (±55.0)	42.7 (±35.99)	52.6 (±64.5)	85.9 (±104.9)	108.8 (±148.0)	111.6 (±87.8)	**0.04**
**Years Worked (%)**										**0.02**
0–1	4 *(40.0%)*	3 *(23.1%)*	2 *(18.2%)*	4 *(40.0%)*	14 *(38.9%)*	5 (*45.5%)*	4 *(16.7%)*	5 *(19.2%*)	1 *(9.1%)*	
2–5	6 *(60.0%)*	6 *(46.2%)*	6 *(54.5%)*	3 *(30.0%)*	11 *(30.6%)*	3 *(27.3%)*	9 *(37.5%)*	16 *(61.5%)*	2 *(18.2%)*	
6–10	0 *(0%)*	3 *(23.1%)*	3 *(27.3%)*	1 *(10.0%)*	8 (*22.2%)*	2 *(18.2%)*	7 *(29.2%)*	4 *(15.4%)*	3 *(27.3%)*	
11–15	0 *(0%)*	1 *(7.7%)*	0 *(0%)*	2 *(20.0%)*	1 *(2.8%)*	0 *(0.0%)*	4 *(16.7%)*	1 (*3.8%)*	3 (*27.3%)*	
16 or more	0 *(0%)*	0 *(0%)*	0 *(0%)*	0 *(0%)*	2 (*5.6%)*	1 *(9.1%)*	0 *(0.0%)*	0 (*0%)*	2 *(18.2%)*	
**Highest Degree Earned (%)**										**< 0.001**
Certificate	0 *(0%)*	0 *(0%)*	0 *(0%)*	0 *(0%)*	4 *(11.1%)*	1 *(9.1%)*	1 *(4.2%)*	0 *(0%)*	0 *(0%)*	
Diploma	6 *(60.0%)*	9 *(69.2%)*	9 (81.8%)	10 *(100.0%)*	29 *(80.6%)*	8 *(72.7%)*	17 *(70.8%)*	16 (*61.5%)*	3 *(27.3%)*	
Bachelors	4 *(40.0%)*	1 *(7.7%)*	2 (18.2%)	0 *(0%)*	3 *(8.3%)*	2 *(18.2%)*	4 (*16.7%)*	8 *(30.8%)*	2 *(18.2%)*	
Masters	0 *(0%)*	3 *(23.1%)*	0 *(0%)*	0 *(0%)*	0 *(0%)*	0 *(0%)*	2 *(8.3%)*	2 *(7.7%)*	6 *(54.5%)*	
Doctorate or more	0 *(0%)*	0 *(0%)*	0 *(0%)*	0 *(0%)*	0 *(0%)*	0 *(0%)*	0 *(0%)*	0 *(0%)*	0 *(0%)*	

Clusters are listed left to right in order of increasing level of facility and numbers of facilities, from Burnt Forest having only two lower level facilities to MTRH, with three tertiary facilities corresponding to three specialist clinics. Comparisons were made by ANOVA for continuous variables and a chi-squared for categorical variables. Statistical significance set at *p* < 0.05

*MTRH* Moi Teaching and Referral Hospital, *MO* Medical Officer, *CO* Clinical Officer, *SD* Standard deviation

In order to assess for interruptions in the referral process, we examined the proportion of providers who responded “None” and/or “Unspecified.” Of the 152 providers interviewed, four (3%) providers reported not referring patients to anyone up the health system, while 69 (45%) did not report down-referrals. Of the 84 providers who did refer patients down, 8 were at tertiary care levels, making up 80% of the 10 total tertiary care providers interviewed. At the secondary level, 43 of a total 72 interviewed (60%) referred down, and 33 of 70 (47%) at primary care levels. Fifty-two of the 152 (34%) providers stated that they refer patients up to unspecified persons and 29 of 152 (19%) providers stated they refer patients down to unspecified persons.

We analyzed each cluster network at the node level to look for individual influencers and bridge-makers. The providers with the three highest in-degree and normalized betweenness scores for each cluster are listed in Table 2. While there was at least one individual who stood out as having the highest in-degree score for up-referrals, only Kocholya, Kitale, and Turbo clusters had any individuals with in-degree scores greater than 1 for down-referrals. Normalized betweenness scores for individuals were greater for up-referrals compared to down-referrals in all clusters except Butula, Mosoriot, and Turbo.

**Table 2:** Node level scores by cluster

	Burnt Forest		Webuye/Bungoma		Butula		Mosoriot/Nandi		Kocholya/Busia		Bunyala		Turbo		Kitale/Trans Nzoia		MTRH	
	ID^**a**^	Score	ID	Score	ID	Score	ID	Score	ID	Score	ID	Score	ID	Score	ID	Score	ID	Score
**Highest In-Degree**																		
*Referral Up*	306	2	272	5	406	5	201	4	177	11	428	3	252	8	142	19	219	7
	84	1	275	3	75	2	251	2	98	7	431	3	222	5	91	4	222	6
	111	1	81	3	74	2	222	2	99	4	406	1	263	4	139	3	220	6
*Referral Down*	118	1	275	1			194	1	173	5	428	1	240	4	156	2	226	1
	117	1	184	1			190	1	168	4	431	1	242	3	138	1	218	1
			273	0			191	1	186	3	406	1	252	3	149	1	142	1
**Highest Betweenness**																		
*Referral Up*	111	0.07	273	0.06	431	0.01	201	0.13	289	0.08	406	0.05	251	0.09	139	0.05	219	0.07
	103	0.06	275	0.05	382	0.01	192	0.01	172	0.05	403	0.02	262	0.07	158	0.01	220	0.05
	117	0.03	274	0.03					169	0.05	426	0.01	261	0.01	293	0.01	371	0.05
*Referral Down*	111	0.03	275	0.03	379	0.01	201	0.2	176	0.04	428	0.04	252	0.18	139	0.02	219	0.07
	306	0.03	273	0.03					179	0.02	406	0.04	251	0.16			373	0.02
			274	0.01					172	0.02			242	0.14			226	0.01

^a^Anonymous provider identification number

Regression results shown in Table 3 demonstrated that higher levels of training, higher facility levels, male sex, and greater number of years working were predictive of receipt of referral for complicated or uncontrolled hypertension. Specifically, consultants were 6.5 (95% CI 2.7–16.3) times as likely to receive an up-referral than clinical officers (*p* < 0.01). County hospitals were 26.9 (95% CI 6.0–119.3) times as likely to receive referrals compared to primary centers. Sub-county hospitals were 2.5 (95% CI 1.3–2.6) times as likely to receive referrals compared to primary centers. Individuals who had worked > 11 years at a facility were 4.4 times as likely (*p* < .01) and 6–10 years were also 4.4 times as likely (*p* < 0.1) as an individual who was in their first year to receive a referral. Men were 2 (95% CI 1.2–3.8) times as likely to receive referrals than women (*p* < 0.05).

**Table 3:** Relationship between provider characteristics and likelihood of receiving a referral up the health system

Provider Characteristics	IRR	95% CI	*P*-value
Provider Role			
Consultant	6.5	2.6–16.3	0.00
Medical Officer	1.9	1.0–3.7	0.04
Nurse	0.2	0.1–0.5	0.03
Facility Level			
County hospital	26.9	6.0–119.3	0.00
Sub-county hospital	2.5	1.3–4.6	0.02
Years worked at facility			
> 11 yrs. at facility	4.4	1.6–12.2	0.00
6–10 yrs. at facility	4.4	1.7–11.9	0.00
2–5 yrs. at facility	2.4	1.1–5.1	0.02
Sex			
Male	2.3	1.4–3.9	0.00
Avg # HTN Patients/ Month	1.3	1.0–1.7	0.09
Age	0.9	0.9–1.0	0.01

Comparisons were made using Mixed-Effects Poisson Regression Model between centrality scores and likelihood of receiving a referral up the health system as calculated by the incidence rate ratio due to non-normal distribution of the dependent variable. Reference values are as follows: Sex, female; Years Worked, 0–1 year; Title, Clinical Officer; Facility Level, Health Centre + Dispensary. Statistical significance set at *p* < 0.05

*HTN* Hypertension, *IRR* Incidence rate ratio

**Fig. 2:**
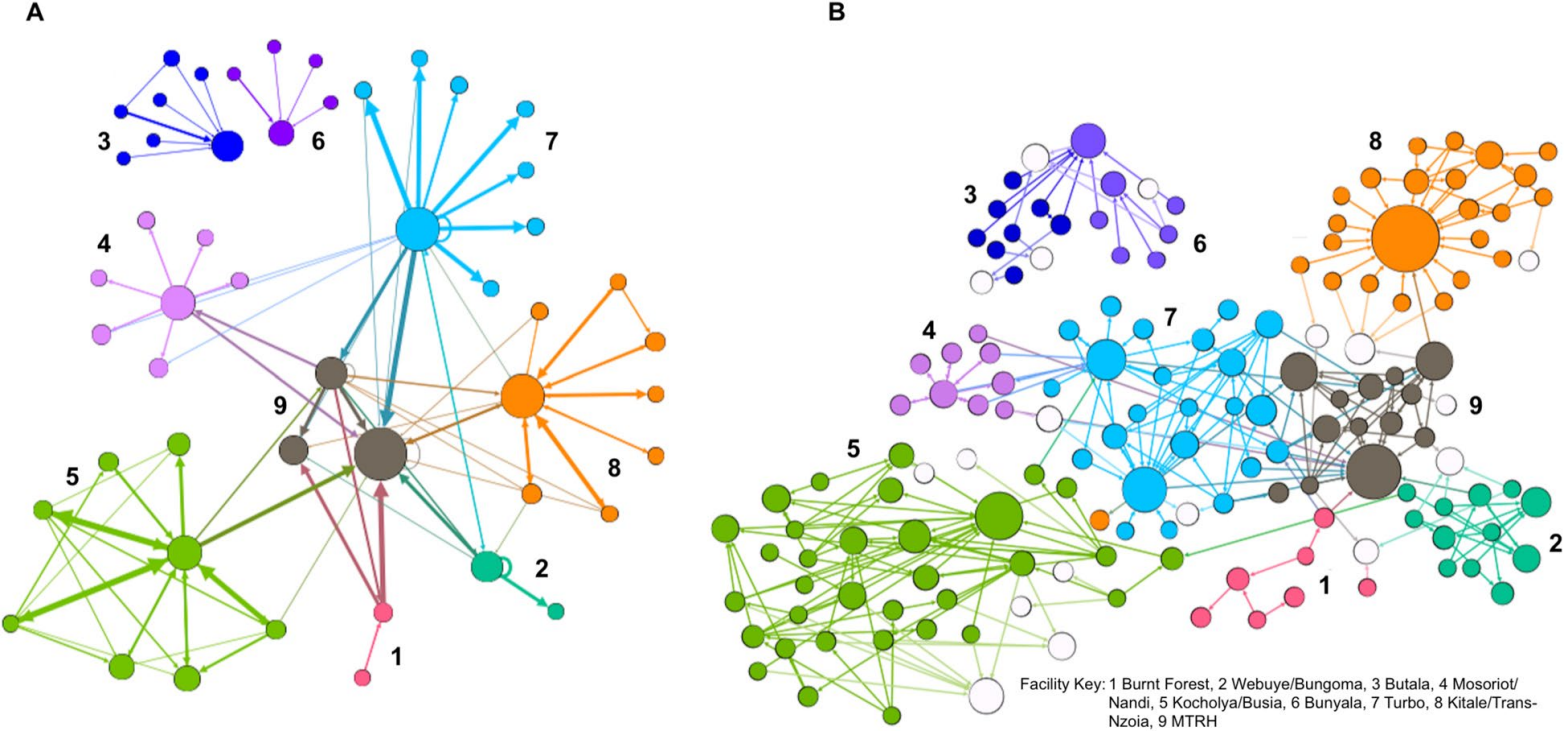
Facility (**A**) and Provider (**B**) Level Networks. Nodes are colored by geographic cluster. The size of each node represents in-degree nominations: size increase proportionally with nominations. Thicker edges (Arrows) demonstrate a greater number of connections between specific nodes. Panel **A** shows the facility referral network model and Panel **B** shows the provider referral network model

Table 4 demonstrates the CP scores for the provider and facility level networks. The CP scores for provider networks range from 0.335 to 0.693, indicating less correlation with a perfect referral system while the CP scores for the facility networks range from 0.707 to 0.949 which indicate a more highly integrated referral network. Of the provider networks, Mosoriot and Bunyala had the highest CP scores, 0.693 and 0.615 respectively. Figure 2 shows a visual representation of the strengths of these referral networks. Each node represents a facility (2A) or provider/respondent (2B), while the lines between two nodes represent a referral. Each arrow indicates target of a referral from a specific source or node.

**Table 4:** Core periphery scores by cluster

	Burnt Forest	Webuye/Bungoma	Butula	Mosoriot/Nandi	Kocholya/Busia	Bunyala	Turbo	Kitale/Trans Nzoia	MTRH
**Provider Referrals**	0.433	0.407	0.463	0.639	0.335	0.615	0.449	0.424	0.535
**Facility Referrals**	0.949	0.857	0.707	0.949	0.871	0.707	0.904	0.894	0.949

## Discussion

This social network analysis examined baseline hypertension referral networks between individual providers and facilities across nine geographic clusters in western Kenya. Our analysis highlighted potential challenges in weakness in referral networks for hypertension, including: cluster variability in characteristics, poor provider linkages, and the lack of a cohesive down-referral system. These challenges represent opportunities focused on creating and implementing interventions to improve referral networks for hypertension.

Understanding the heterogeneity in demographics across the clusters is crucial to understand the data, as networks are highly susceptible to external factors including local politics, land topography, and/or economy. The differences in provider level, highest degree earned, and years worked at facility were likely driven by the types of facilities that made up each cluster. Clusters which had more secondary and tertiary facilities also had more providers with higher training levels, such as physicians and medical officers, and providers with greater than 6 years’ experience compared to clusters with primary facilities which were predominantly run by clinical officers and nurses. Another underlying difference may be geography as secondary and tertiary facilities are usually located in urban city centers, while primary facilities are often in rural sites which have trouble retaining providers and are at high risk of provider turnover [47].

Despite the variability in clusters, our core periphery models showed strong referral networks in place at the facility level, though these networks were less structured when analyzed at the provider level. We hypothesize that the weaker provider networks are a result of providers not knowing to whom they are sending patients because of distance or turnover, and because there is not a structured method for specifying providers during referral. It is important to distinguish that weak provider networks do not mean that patients are not completing the referrals, but rather it indicates that referral completions are likely not stemming from provider-to-provider relationships. Based on social network theory and correlation between social capital and health in LMICs, we would hypothesize that clusters like Mosoriot and Bunyala with higher provider core periphery scores would have higher rates of referral completion by garnering more trust with patients in this context [48, 49]. Since referral completion rates have not been systematically documented across sites, we were unable to explore these hypotheses at this time, but referral completion and other referral process metrics are secondary outcomes of the STRENGTHS trial.

Provider networks followed a predictable pattern for referring patients up to facilities where individuals had more training, higher level facilities, and more years of work experience. There were insufficient data to observe patterns for down-referrals because a majority of providers across all levels stated they do not refer patients with controlled hypertension down to lower facilities. The lack of a cohesive down-referral system was also shown in nodal analysis as most clusters were less likely to have individuals with higher centrality scores in their down-referral compared to up-referral networks. This finding was concerning due to the stated goal of the Kenyan Ministry of Health in their 2014–2018 Referral Strategy to create effective networks both up and down the health system [16]. Anecdotally, providers at higher level facilities were concerned that the lack of down-referrals leads to patients spending unnecessary resources to get to higher level facilities creating bottlenecks, increased waiting times, and medication stockouts when patients could have been treated at a lower level. Demographic data showed that providers from MTRH and Kitale - clusters with more secondary and tertiary facilities - saw higher numbers of patients with hypertension on average. This comparison is limited, however, by large confidence intervals and that Burnt Forest also had a higher average number without having proportionally more high-level facilities. Of note, male providers were two times as likely to receive a referral than women providers. This could be due to perceived competency of men versus women as providers, or due to the fact that 75% of upper level providers (medical officers and physicians) were men. To better understand these results, it will be important to correlate these findings with qualitative discussions of gender roles and to follow up whether or not this trend persists after our intervention.

Prior research utilizing social network analysis for referrals in LMICs have been limited and heterogenous in approach. Thomas et al. [50] examined network density for referrals to HIV services and family planning in Addis Ababa, demonstrating that a network with increased links and higher density was correlated with more patients reporting referrals and that their needs were met. The current study supports the hypothesis that greater provider connections may lead to more referral completions, but it reinforces the need for referral completion data in order to better understand this relationship in Western Kenya. The current study has limitations that must also be considered. We were unable to interview all providers in the STRENGTHS referral network. In an effort to capture a more robust referral system, we included sites across Western Kenya which were geographically distant from one another. Due to time and resource constraints, complete data collection for all providers at a particular location sometimes had to take place over 1 to 2 days. In order to mitigate these constraints, site administrators were asked to have all providers who worked at a site present on the days of data collection. However, some providers were unable to be sampled as they were on vacation, working at a different location or were otherwise unavailable. In order to capture referral patterns, providers *who worked in multiple sites* were asked to respond to the survey based on where they conducted the majority of their clinical practice to capture the most common referral patterns. Both limitations led to missing referral data, a common challenge in social network analysis, although degree centrality is robust to missing [51, 52]. Future iterations of such research may benefit from a more limited geographic space to better capture all individuals in the referral network; however, this would limit the relevance within the greater healthcare system. Our results were further limited by a small sample size of providers within each cluster which led to greater variability in the analysis. This, compounded by heterogeneity across clusters, limited the applicability of the results beyond this region.

Providers at all levels of the health care system receive training on hypertensive referral algorithms. Despite training and written guidelines, not all clinicians choose to refer their patients. The referral network analysis, detailed in this paper, was structured to quantitively capture the effectiveness of such referral guidelines*.* These results provided useful insights into the creation of our STRENGTHS referral network intervention. First, we used the results of our node level analysis to identify specific influencers and linkers who could join the Human Centered Design team as community champions to help build our intervention which includes peer navigation and health information technology [24, 25]. Health systems are quite variable in how they may prove effective. A concurrent qualitative analysis conducted by the STRENGTHS team, demonstrated a strong sense of loyalty and trust between patients and providers as well as amongst colleagues. With this context in mind, it is plausible that a strong provider connection may assist with the initiation of referral and completion of referrals. Thus, we have specifically targeted aspects of our intervention to dictate referral steps for both up- and down-referrals such as indicating the referring provider, acknowledgement of a referral completion by the receiving provider, as well as the referral facility names; all components that were previously not recorded. The network analysis highlighted that providers are less likely to refer down, so we have tailored our trainings and educational materials for both peer navigators and providers to emphasize the importance of down-referrals when patients have stabilized. We plan to reexamine these networks using both core-periphery models and regression upon completion of the intervention to better understand relationships between provider networks, facility networks, and referral completion.

## Conclusions

Effective referral systems for the care of patients with hypertension must acknowledge and analyze both provider and facility characteristics. By using network analysis among nine clusters of healthcare facilities in western Kenya, we identified that heterogeneity in provider characteristics, poor provider-to-provider linkages, and lack of an organized down-referral system were important determinants of the strength of the referral system. Network analysis is an effective method to understand these components of the referral system for chronic conditions like hypertension and for designing network informed interventions. These findings are being used to design the intervention package of approaches in the STRENGTHS study to improve outcomes for patients with hypertension. This study also has greater implications for the management of chronic diseases globally by offering a network analysis framework for clinicians and researchers looking to understand and intervene on the challenges of managing chronic disease across a health system and different contexts worldwide.

## Supplementary Information

**Additional file 1.** STRENGTHS Referral Network Survey.

## Data Availability

The datasets used and analyzed during the current study are available from the corresponding author on reasonable request. All data are stored on a secure server through the AMPATH program and are not shared publicly at this time as the STRENGTHS study is ongoing. Deidentified data from the RNA analysis can be shared.

## Abbreviations

AMPATH: Academic Model Providing Access to Healthcare
CDM: Chronic Disease Management
CP: Core Periphery
CVD: Cardiovascular Disease
HIV: Human Immunodeficiency Virus
LMIC: Low-and-Middle Income Countries
MTRH: Moi Teaching and Referral Hospital
STRENGTHS: Strengthening Referral Networks for Management of Hypertension Across the Health System
