# Supplementary material for: Network characteristics of a referral system for patients with hypertension in Western Kenya: results from the Strengthening Referral Networks for Management of Hypertension Across the Health System (STRENGTHS) study
Source: BMC Health Serv Res. 2022 Mar 7;22:315. doi: 10.1186/s12913-022-07699-8 (PMC8903732; doi:10.1186/s12913-022-07699-8)
Supplement: Supplementary file 1 — Additional file 1. STRENGTHS Referral Network Survey. [file 12913_2022_7699_MOESM1_ESM.docx]

**Additional file for “Network Characteristics of a Referral System for Patients with Hypertension in Western Kenya: Results from the Strengthening Referral Networks for Management of Hypertension Across the Health System (STRENGTHS) Study”**

Aarti Thakkar, Thomas Valente, Josephine Andesia, Benson Njuguna, Juliet Miheso, Tim Mercer, Richard Mugo, Ann Mwangi, Eunice Mwangi, Sonak D. Pastakia, Shravani Pathak, McKinsey M. Pillsbury, Jemima Kamano, Violet Naanyu, Makeda Williams, Rajesh Vedanthan, Constantine Akwanalo, Gerald S. Bloomfield

**STRENGTHS Referral Network Survey**

**Study ID: _______**

**Age: _______**

Q1.

**What is the name of the primary facility where you work?**

Q2.

**How many years have you worked at your current health facility?**

0-1 year

2-5 years

6-10 years

11-15 years

16 or more years

Q3:

**What is your job title?**

Consultant

Physician

Medical Officer

Clinical Officer

Nurse

Other

Q4:

**What is your highest academic degree?**

Certificate

Diploma

Bachelors

Masters

Doctorate or Higher

Q5:

**How many patients in an average month do you see who are hypertensive?**

The first set of questions asks you to select colleagues with whom you communicate about patients with complicated or uncontrolled hypertension. You will have the the opportunity to list as many names as you think appropriate, but no more than 7.

Q6:

**Who are the providers you refer patients with hypertension, or complications of hypertension such as cardiovascular disease to?**

Q7:

**What facilities do you refer patients with hypertension or cardiovascular disease to?**

This set of questions asks you to select colleagues with whom you communicate about patients with benign or controlled hypertension. You will have the the opportunity to list as many names as you think appropriate, but no more than 7.

Q8:

**Who are the providers you refer patients with benign or uncomplicated hypertension to?**

Q9.

**What facilities do you refer patients benign or uncomplicated hypertension to?**

Q10: **Please use the space below to provide any comments or suggestions you have about this survey:**
